# Recurrent Painful Ophthalmoplegic Neuropathy Responding to Lamotrigine: A Case Report

**DOI:** 10.7759/cureus.56924

**Published:** 2024-03-25

**Authors:** Ahmed M Aljammaz, Haya Al Orainni, Azzam I Alnughaythir

**Affiliations:** 1 Vascular Neurology, Medical Cities Program, Ministry of Interior, Riyadh, SAU; 2 Neurology, Security Forces Hospital, Riyadh, SAU

**Keywords:** recurrent painful ophthalmoplegic neuropathy, cranial nerve 6 palsy, lamotrigine, ophthalmoplegic migraine, migraine disorder, adult neurology

## Abstract

Recurrent painful ophthalmoplegic neuropathy (RPON) is a rare neurological disorder characterized by recurring ipsilateral headache and paresis of one or more ocular motor nerves. We report the case of a 56-year-old woman with systemic lupus erythematosus (SLE) and hypertension, who presented with severe recurring headaches, nausea, and vomiting. Initially misdiagnosed with cerebral venous sinus thrombosis, her symptoms persisted despite anticoagulant therapy. Further evaluation led to the diagnosis of RPON. Management included intravenous analgesia, hydration, and indomethacin for pain relief. Persistent headache episodes necessitated the introduction of lamotrigine, resulting in significant symptom improvement. However, discontinuation of lamotrigine led to a recurrence of symptoms, which resolved upon resuming the medication. This case contributes to the limited RPON literature, providing insights into its diagnosis and management, with the goal of enhancing awareness and improving patient care.

## Introduction

Recurrent painful ophthalmoplegic neuropathy (RPON), once known as ophthalmoplegic migraine, is an extremely uncommon condition. The diagnosis is made when at least two attacks of unilateral headache and ipsilateral ophthalmoplegia are experienced [1]. An estimated 0.7 per million individuals are affected annually, making the diagnosis more difficult [2]. The International Classification of Headache Disorders 3rd edition (ICHD-3) established the criteria for diagnosis and considered that the disorder is recurring painful neuropathy rather than a migraine variant, which is why the term was revised [1]. The oculomotor nerve is most frequently affected, with mydriasis and ptosis being the most common manifestations, followed by abducent and trochlear nerve [3]. Magnetic resonance imaging (MRI) with gadolinium administration has identified changes, like contrast enhancement or nerve thickening, during attacks [4]. Although the disorder mainly affects children, there have also been reports in older individuals, with an average age of eight years old [4]. Some patients can benefit from the use of corticosteroids [1]. On the other hand, lamotrigine was used in this case as there is evidence that supports using it as a preventive medication for migraine with aura [5]. This case report documents a unique case of RPON in order to contribute to the existing literature and provide insights into the diagnosis and management of this condition. By shedding light on this rare condition, we aim to enhance understanding, facilitate early and accurate diagnosis, and explore potential treatment options for the benefit of patients suffering from RPON.

## Case presentation

A 56-year-old woman, with a known case of hypertension and systemic lupus erythematosus (SLE), presented to the emergency department with complaints of moderate to severe headache associated with nausea and vomiting. The patient reported a two-week history of intermittent unilateral headaches associated with ipsilateral diplopia, which were initially diagnosed as cerebral venous sinus thrombosis at a peripheral hospital. She had been receiving low-molecular-weight heparin (LMWH) therapy. However, her symptoms continued, and no improvement or side effects were noted from the LMWH treatment. This prompted her to seek care at our emergency room. The patient described her headaches as unilateral with left-sided dullness. She reported associated symptoms of nausea, vomiting, facial puffiness, and photophobia. There was no history of fever, contact with sick patients, or consumption of unpasteurized milk. The headache only responded to intravenous analgesia. The patient denied any recent trauma or significant medical events. Her medical history was significant for hypertension, which was managed with medications. Upon presentation to the emergency room, the patient was in moderate distress due to the headache. Vital signs, including blood pressure, heart rate, respiratory rate, and oxygen saturation, were within normal limits. Neurological examination revealed a fully alert and oriented patient with normal speech and cognitive function. Cranial nerve examination revealed no abnormalities, including assessment of visual acuity, pupillary reflexes, extraocular movements, and fundoscopic examination. Motor strength, sensation, and coordination were intact in all extremities. There were no signs of meningeal irritation, such as neck stiffness or Kernig's sign. The rest of the systemic examination, including cardiovascular, respiratory, and abdominal examinations, was unremarkable.

**Figure 1:**
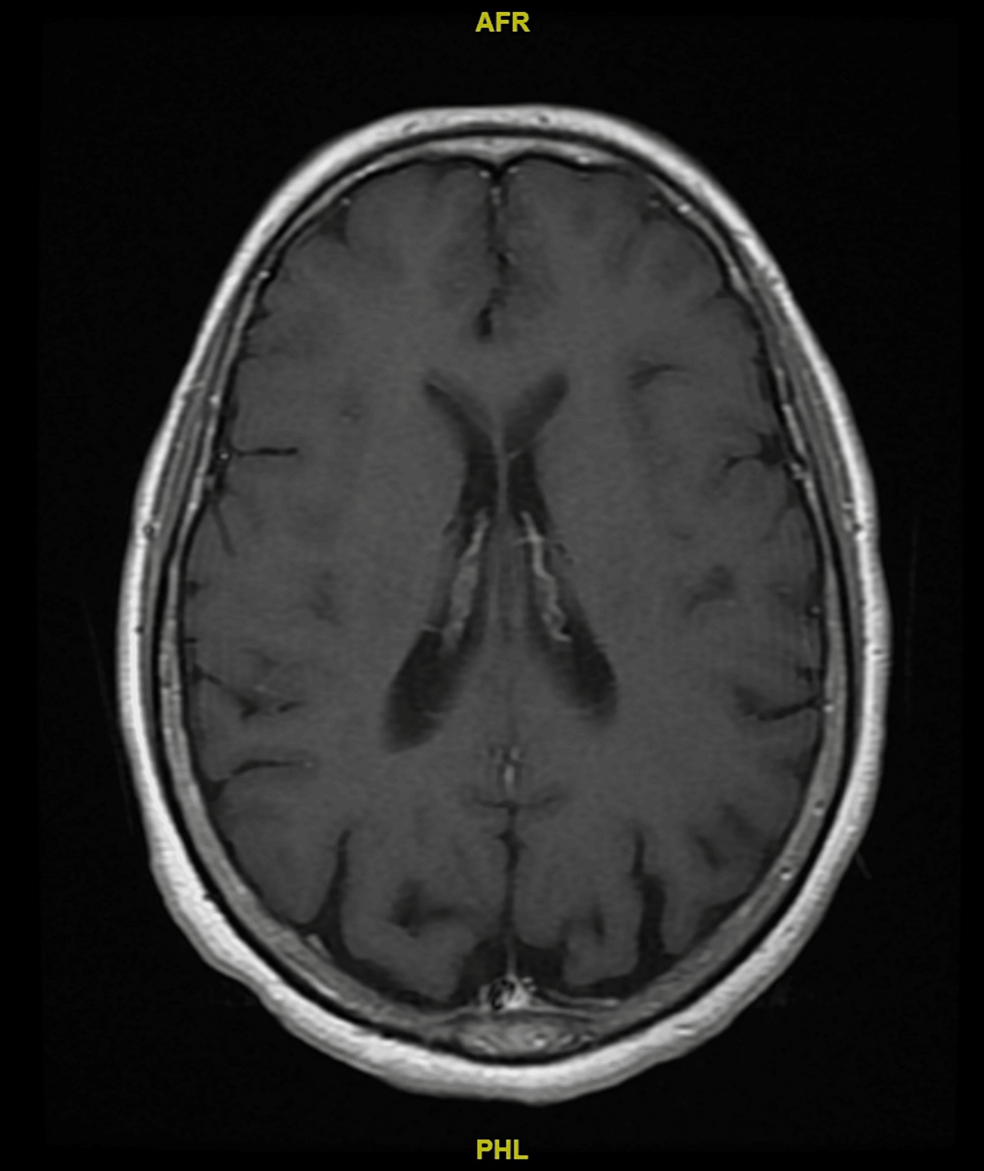
Non-contrast cerebral MRI showing dilatation of CSF spaces more than expected for the patient's age and nonspecific white matter changes in both cerebral hemispheres MRI: magnetic resonance imaging, CSF: cerebrospinal fluid

**Figure 2:**
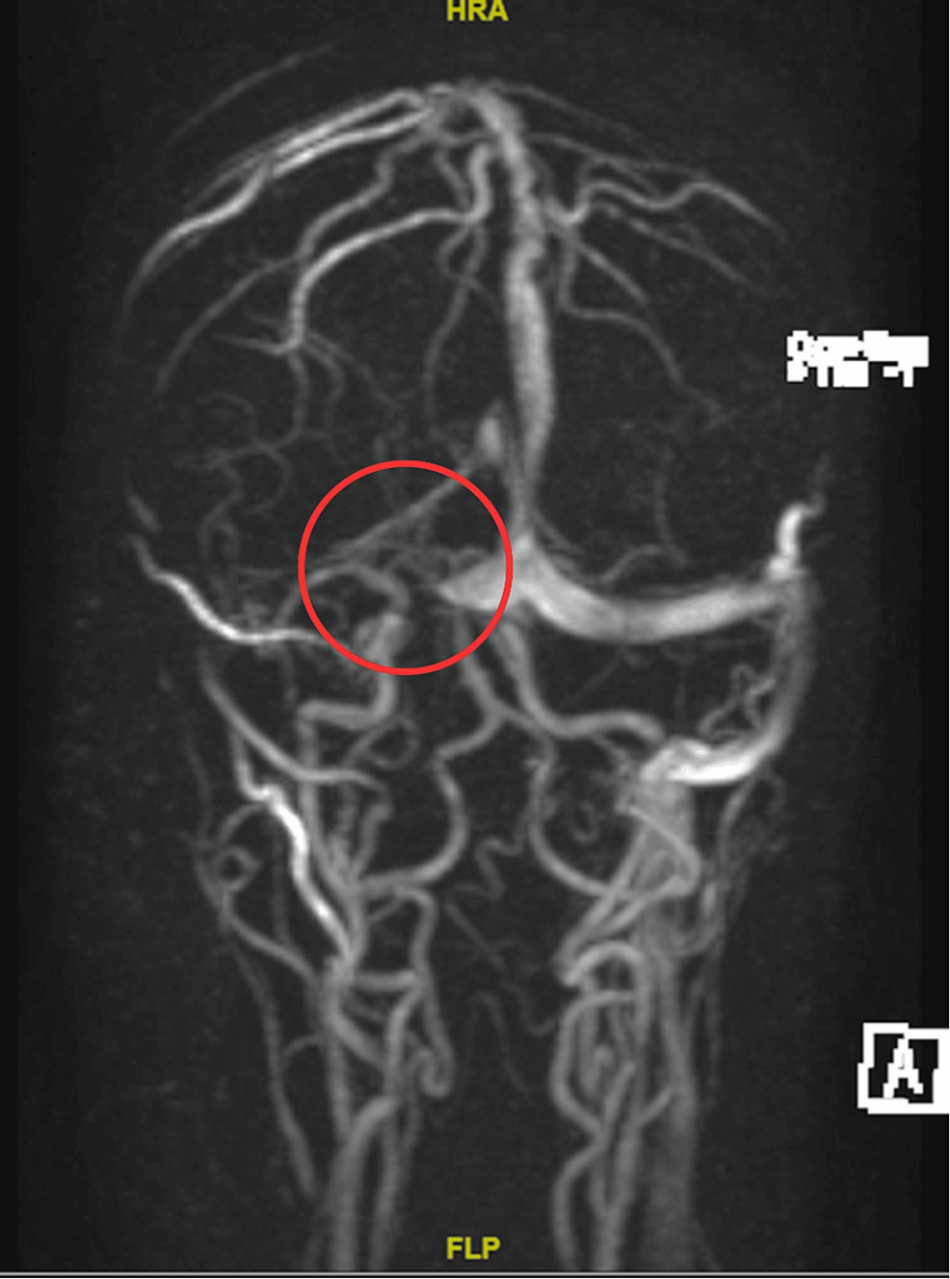
Cerebral MRV showing hypoplastic right transverse and sigmoid sinuses with a normal caliber of the other dural sinuses MRV: magnetic resonance venography

The next morning after admission to our emergency room, the patient developed diplopia and sixth cranial nerve palsy. Based on the history and physical examination findings, a diagnostic evaluation was initiated, including neuroimaging studies and laboratory investigations, which confirmed the diagnosis of RPON and ruled out other potential causes, such as cerebral venous sinus thrombosis. A series of diagnostic tests were performed to confirm the diagnosis and exclude other potential causes. Neuroimaging studies included a non-contrast cerebral MRI and cerebral magnetic resonance venography (MRV). The CT venography revealed multiple small nodular filling defects in the right transverse sinus, which were suggestive of pacchionian granulation tissue, which was done by the peripheral hospital and reviewed by the neuroradiologist. The non-contrast cerebral MRI showed dilatation of cerebrospinal fluid spaces beyond what is expected for the patient's age and nonspecific white matter changes in both cerebral hemispheres (Figure 1). Lastly, cerebral MRV was reported as hypoplastic right transverse and sigmoid sinuses with normal caliber of the other dural sinuses (Figure 2). Laboratory investigations were also conducted to assess for any underlying systemic conditions that could contribute to the patient's symptoms. Routine lab tests, including a complete blood count, comprehensive metabolic panel, and coagulation profile, were unremarkable. However, the autoimmune panel revealed positive antinuclear antibody (ANA) and anti-double-stranded DNA (anti-dsDNA) antibodies.

During the patient's admission, the acute symptoms, including the severe headache and associated symptoms, were managed with intravenous paracetamol for pain relief and adequate hydration. The patient's headache improved significantly, and upon discharge, she was started on indomethacin for further pain management. The patient was seen by rheumatology and diagnosed with SLE, based on the presence of arthritis, discoid rash, and positive ANA and anti-dsDNA antibodies. The patient was initiated on hydroxychloroquine, a commonly used medication in the management of SLE. During the four-week neurology follow-up, the patient reported persistent headaches and an increased reliance on non-steroidal anti-inflammatory drugs (NSAIDs) for symptom relief. As a result, a decision was made to gradually introduce lamotrigine, an antiepileptic medication with potential efficacy in RPON. The lamotrigine dosage was titrated up to 50 mg every 12 hours over time. In the six-month follow-up, the patient reported feeling significantly better with a reduction in headache frequency and severity. Interestingly, after three months of lamotrigine treatment, the patient decided to discontinue the medication on her own accord. During this time, she experienced three headache attacks without the presence of ophthalmoplegia. The patient was educated about the importance of prophylactic treatment for RPON and the potential benefit of continuing lamotrigine. Consequently, she was resumed on lamotrigine at a dosage of 50 mg twice daily to maintain symptom control. Throughout the follow-up period, the patient underwent regular monitoring and assessment. Neurological examinations did not reveal any new abnormalities, and the patient reported a significant improvement in her quality of life with a reduction in headache frequency and severity.

## Discussion

RPON is an uncommon neurological disorder characterized by recurrent ipsilateral headache and potential weakness or paralysis of one or more ocular motor nerves. Symptom onset ranges from immediate to up to two weeks following the initial headache. Ophthalmoplegia can persist for weeks to months but generally resolves [4]. The current case outlines an atypical headache presentation, presenting diagnostic challenges. The patient in question arrived at the emergency department with a severe, recurrent headache lasting two weeks, which was followed by sixth cranial nerve palsy and diplopia after one day. A retrospective clinical study conducted in China over four years, including eight patients, revealed that 75% experienced third nerve symptoms, like mydriasis and ptosis. Sixth cranial nerve palsy was observed in 50% of cases, while fourth nerve involvement was less common at 12.5% [6]. Radiological findings in RPON are diverse, ranging from normal MRIs with gadolinium and nerve enhancement, particularly of the third nerve, to vascular images that often show no significant abnormalities [4]. In the case presented here, CT venography identified pacchionian granulation tissue in the right transverse sinus, which was initially mistaken for venous thrombosis at another facility. Laboratory results were mostly normal, except for the presence of ANAs and anti-double-stranded DNA antibodies.

The literature does not establish a definitive link between positive ANA, dsDNA, and RPON. However, it has been noted that headaches, including migraines, affect approximately one-third of patients with SLE [7]. The question arises whether there is an association between RPON and SLE. In the acute phase, the patient did not receive corticosteroids; instead, treatment involved intravenous analgesia and hydration. This diverges from other reports where corticosteroids are commonly used in acute management, usually resulting in symptom resolution. Prophylactic treatments are less frequently documented [4,6,8]. In this instance, the patient responded well to a six-month course of lamotrigine. Another case involving a 40-year-old male with left-sided ophthalmoplegia and headache reported successful prophylaxis using propranolol and flunarizine over one year [9]. In addition, positive outcomes were achieved with pregabalin at a dose of 150 mg daily for one year in a patient who did not respond to beta-blockers, calcium channel blockers, topiramate, or tricyclic antidepressants [8]. The literature review indicates that steroids, lamotrigine, propranolol, flunarizine, and pregabalin are potential management options for RPON. There is a need for more comprehensive clinical trials to establish the most effective treatment protocols for this condition. To summarize, while steroids are effective for acute RPON management, alternative medications, such as lamotrigine, propranolol, flunarizine, and pregabalin, have been associated with favorable outcomes for symptom relief and prophylaxis.

## Conclusions

RPON is a rare condition that can present with challenging diagnostic dilemmas. This case demonstrates the complexities involved in the diagnosis and management of RPON. It also highlights the potential benefits of lamotrigine as a preventive medication for this condition. Further research and treatment trials are required to enhance our understanding of this condition and optimize patient care. This case highlights the diagnostic challenges associated with RPON and emphasizes the importance of considering this condition when a patient presents with recurrent headaches and unilateral ophthalmoplegia. Further clinical trials and investigations are needed to establish effective treatment strategies and improve patient outcomes.

## References

[1] Gobel H (2023). IHS Classification ICHD-13: 13.10 Recurrent painful ophthalmoplegic neuropathy. https://ichd-3.org/13-painful-cranial-neuropathies-and-other-facial-pains/13-10-burning-mouth-syndrome-bms/.

[2] Hansen SL, Borelli-Møller L, Strange P, Nielsen BM, Olesen J (1990). Ophthalmoplegic migraine: diagnostic criteria, incidence of hospitalization and possible etiology. Acta Neurol Scand.

[3] Lane R, Davies P (2010). Ophthalmoplegic migraine: the case for reclassification. Cephalalgia.

[4] Gelfand AA, Gelfand JM, Prabakhar P, Goadsby PJ (2012). Ophthalmoplegic "migraine" or recurrent ophthalmoplegic cranial neuropathy: new cases and a systematic review. J Child Neurol.

[5] Smeralda CL, Gigli GL, Janes F, Valente M (2020). May lamotrigine be an alternative to topiramate in the prevention of migraine with aura? Results of a retrospective study. BMJ Neurol Open.

[6] Li C, Huang X, Tan X, Fang Y, Yan J (2021). A clinical retrospective study of recurrent painful ophthalmoplegic neuropathy in adults. J Ophthalmol.

[7] Sarangi S, Nahak SK, Rupashree A, Panigrahi J, Panda AK (2023). Prevalence of migraine in systemic lupus erythematosus: a meta-analysis. Lupus.

[8] Zamproni LN, Ribeiro RT, Cardeal M (2019). Treatment of recurrent painful ophthalmoplegic neuropathy: a case where pregabalin was successfully employed. Case Rep Neurol Med.

[9] Alam A, Iqubal MS, Kumar B, Azad ZR (2021). A case of adult-onset ophthalmoplegic migraine. Neurol India.

